# Structural Probing with MNase Tethered to Ribosome Assembly Factors Resolves Flexible RNA Regions within the Nascent Pre-Ribosomal RNA

**DOI:** 10.3390/ncrna8010001

**Published:** 2022-01-09

**Authors:** Tom Dielforder, Christina Maria Braun, Fabian Hölzgen, Shuang Li, Mona Thiele, Marina Huber, Uli Ohmayer, Jorge Perez-Fernandez

**Affiliations:** Department of Biochemistry III, University of Regensburg, Universitätstrasse 31, D-93051 Regensburg, Germany; Tom.Dielforder@vkl.uni-regensburg.de (T.D.); Christina.Braun@vkl.uni-regensburg.de (C.M.B.); hoelzgen@post.bgu.ac.il (F.H.); shuang.li@biogents.com (S.L.); mona.christin.thiele@gmail.com (M.T.); marina-huber1@web.de (M.H.); uli.ohmayer@gmx.de (U.O.)

**Keywords:** ribosome biogenesis, SSU-processome, *S. cerevisiae*, structural probing, RNA

## Abstract

The synthesis of ribosomes involves the correct folding of the pre-ribosomal RNA within pre-ribosomal particles. The first ribosomal precursor or small subunit processome assembles stepwise on the nascent transcript of the 35S gene. At the earlier stages, the pre-ribosomal particles undergo structural and compositional changes, resulting in heterogeneous populations of particles with highly flexible regions. Structural probing methods are suitable for resolving these structures and providing evidence about the architecture of ribonucleoprotein complexes. Our approach used MNase tethered to the assembly factors Nan1/Utp17, Utp10, Utp12, and Utp13, which among other factors, initiate the formation of the small subunit processome. Our results provide dynamic information about the folding of the pre-ribosomes by elucidating the relative organization of the 5′ETS and ITS1 regions within the 35S and U3 snoRNA around the C-terminal domains of Nan1/Utp17, Utp10, Utp12, and Utp13.

## 1. Introduction

**Figure 1:**
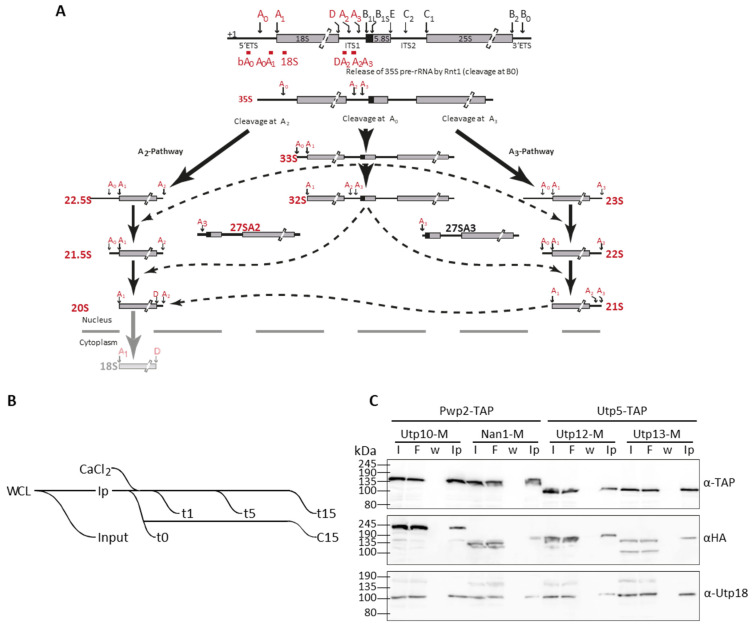
Processing of rRNA and structural probing assays. (**A**) Scheme for the processing of pre-rRNA to produce the 18S rRNA. Alternative cleavage pathways were described [8]. (**B**) Experimental workflow. Whole-cell lysates (WCL) were obtained for affinity purification (Ip). A fraction of WCL was used as input fraction for protein and RNA analyses. Affinity purified samples were incubated in the presence of Ca^2+^ to activate MNase activity for the indicated time. Two aliquots of samples non-treated with Ca^2+^ were collected as control samples, t0 before Ca^2+^ addition and c15 after 15 min of incubation in the absence of Ca^2+^. (**C**) Different affinity purification fractions were analyzed by western blotting with indicated antibodies (anti-TAP, anti-HA, and anti-Utp18). I, input fraction from WCL; F, flow-through or unbound fraction to the affinity matrix; w, last wash fraction; Ip, affinity-purified samples.

Ribosome biogenesis is an essential process for cell biology. In eukaryotic cells, the ribosomal RNAs (rRNAs) 18S, 5.8S, and 25S form part of a single pre-rRNA (35S), and they are separated by flanking sequences absent in the mature ribosomes (Figure 1A). These flanking sequences are removed during the maturation of the pre-ribosomal particles. More than 200 assembly factors (AFs) participate in the pre-ribosomal particles’ maturation, a process including the stable incorporation of ribosomal proteins, modification of nucleotides, removal of flanking sequences, and folding of the pre-rRNA.

Several AFs associate with the nascent pre-rRNA to form the earliest identified pre-ribosomal precursor [1,2,3]. Formation of the first precursor of the small ribosomal subunit (SSU) or SSU-processome is a stepwise process driven by the hierarchical relationships between different AFs and folding of the pre-rRNA [4,5,6,7]. In this regard, some AFs might function as platforms for the subsequent assembly of other AFs [8]. Alternatively, the association of AFs and the subsequent folding of pre-rRNA might build the binding site for AFs and ribosomal proteins [9]. In any case, the correct processing and folding of the 18S rRNA (the rRNA component of the SSU) require the formation of the SSU-processome, which initiates co-transcriptionally. Nevertheless, at least one-third of the 35S transcripts are processed post-transcriptionally in the so-called 90S pre-ribosomes [1,2].

Structural probing methods are a valuable tool used to investigate the structure of heterogeneous ribonucleoparticles. These methods were first used to establish the secondary structures for the 16S rRNA in bacteria and the 18S rRNA in eukaryotes [10,11]. Additionally, the secondary structures of spacer regions flanking the 18S were initially resolved by structural probing methods [12,13]. Structural probing and derived methods have also been crucial to defining the secondary structures of the U3 snoRNA [14,15], the base-pairing sites for U3 in the pre-rRNA [16], and structural changes in the 5′ETS induced by the association of the U3 snoRNP [17]. Although cryo-EM approaches have provided high-resolution structures of snapshots during ribosome biogenesis, around 30% of the U3 snoRNA, 70% of the 5′ETS, and the complete ITS1 have not been resolved by high-resolution analyses [18,19,20,21,22]. Thus, obtaining information about maturation dynamics or the structure of very early pre-ribosomal remains challenging.

As an alternative, low-resolution methodologies are complementary strategies to solve three-dimensional structures of pre-ribosomal particles. In this regard, the micrococcal nuclease (MNase) tethered to ribosomal proteins or AFs probes the rRNA environment for any given protein [23]. MNase can be expressed in yeast without any deleterious effect because its activity depends on the presence of Ca^2+^. Therefore, MNase fusion proteins with AFs that associate with pre-ribosomal particles might be useful tools for investigating the folding of the pre-rRNA during maturation. In the present work, we investigated the rRNA surrounding t-UTP and UTP-B complexes, as they initiate the formation of the SSU-processome. Both complexes share a similar architecture based in a core-complex and a dissociable heterodimer [8,24]. Moreover, t-UTP and UTP-B complex should accommodate to a variety of different conformations during assembly of the SSU-processome which may participate in the recruitment of AFs and ribosomal proteins [8,9]. In this work, we fused MNase to the C-terminal domain (CTD) of the heterodimer components of t-UTP and UTPB and analyzed the cleavage sites produced after MNase induction. Interestingly, several cleavages occurred in regions of the pre-rRNA not visualized in the high-resolution structures of the SSU-processome, indicating that they might correspond to highly flexible regions. Altogether, our results provide new structural information about the putative localization of several domains of the 35S pre-rRNA, the U3 snoRNA, and the CTD of Utp10.

## 2. Results

### 2.1. Structural Probing in the 5′ETS Region Using Tethered MNase to Utps

Among the 13 proteins present in the complexes t-UTP and UTP-B [24,25,26], we fused MNase to the C-terminal domains of the t-UTP components Utp10 and Nan1/Utp17 (hereafter called Nan1) or the UTP-B components Utp12 and Utp13. The fusion protein contains a ten amino acids linker, which used to be present in most tagging systems [27,28,29].

To increase the sensitivity, we performed structural probing on affinity-purified pre-ribosomal particles. First, pre-ribosomal particles were affinity purified with an assembly factor (AF) fused to the TAP tag [30]. Afterward, we induced the MNase-mediated cleavage by the addition of calcium (Figure 1B). We tagged one component of each complex, t-UTP and UTP-B, to avoid steric hindrance due to the presence of two bulky tags within the same protein complex. Therefore, we affinity-purified the UTP-B component Pwp2 fused to Protein A to enrich pre-ribosomal particles containing MNase fused to either Utp10 or Nan1 (t-UTP components, Supplementary Figure S1A). On the other hand, we affinity-purified the t-UTP component Utp5 tagged to Protein A to enrich pre-ribosomal particles containing MNase fused to either Utp12 or Utp13 (UTP-B components, Supplementary Figure S1B).

Cells were collected during the exponential growth phase, and obtained whole-cell lysates (WCL) were used for affinity purification using IgG Sepharose beads. Affinity purified samples were incubated in the presence of Ca*^2+^* for 15 min, and similar aliquots were collected after 1 (t1), 5 (t5), and 15 (t15) min. Negative controls to analyze MNase independent cleavages were performed in the absence of Ca*^2+^* before the incubation (t0) or after 15 min of incubation (C15) (Figure 1B).

Whole-cell extracts (or Input fractions, I) were resolved in SDS-PAGE together with non-bound material (Flowthrough, F), the last wash before elution (w), and the eluted samples (Ip) and further analyzed by western blotting (Figure 1C). Our results confirmed the purification of the bait proteins (either Pwp2 or Utp5), the MNase containing proteins (Nan1, Utp10, Utp12, and Utp13), and the UTP-B component Utp18 (Figure 1C).

**Figure 2:**
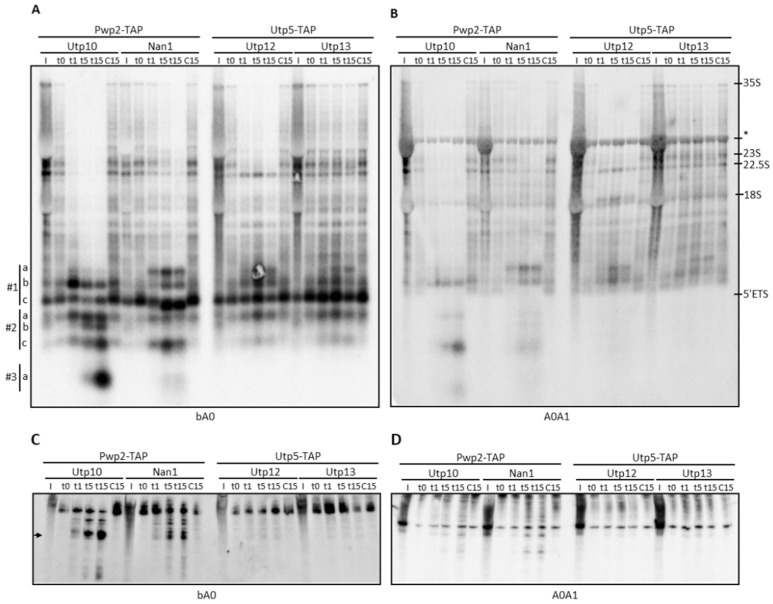
MNase-sensitive sites at the 5′ETS in purified particles. Cleavage patterns were obtained from strains expressing MNase fused to components of the t-UTP/UTP-A and UTP-B complexes. Pre-ribosomal particles containing the MNase fusion proteins were bound to IgG-Sepharose beads. MNase was activated by the addition of 12 µL 0.25 mM CaCl_2_, and samples were incubated at 16 °C during a time course. At indicated time points, 500 µL AE+ buffer was added to stop the reaction. RNAs were obtained by extraction, resolved in formaldehyde–agarose (**A**,**B**) or urea–polyacrylamide (**C**,**D**) gels, and transferred to membranes for northern blot analysis. Hybridizations were performed with oligonucleotides indicated in Table S1 (see also Figure 1A). Components of the UTP complexes fused to TAP or MNase are indicated on top, and probes used for northern blot detection are beside the figure. The localization of some prominent rRNAs is indicated on the right side. The bottom area of the northern blots obtained from agarose gels was classified into three different regions (#1 to #3) to describe the observed RNA fragments. * refers to unspecific 27S band observed.

We analyzed the pre-rRNAs by northern blotting with probes, base pairing at different positions within the 35S pre-rRNA (Figure 1A). In samples purified with the bait proteins Pwp2 and Utp5 and probed at the 5*′*ETS region (oligonucleotides bA0 and A0A1), we observed enrichment of the 35S, 23S, and 22.5S pre-rRNAs, and the 5*′*ETS (pre-rRNA fragment from the transcription start site till the A0 cleavage site) (Figure 2A,B, compare lanes I and t0). After MNase activation, we observed a rapid cleavage of 35S by MNase fused to either Utp10, Nan1, or Utp12 (Figure 2A,B, lanes t1–t15 of Utp10, Nan1, and Utp12 fusion proteins). In contrast, structural probing with Utp13-MNase showed a slower kinetic, and 35S pre-rRNA was only partially cleaved after 15 min of incubation (Figure 2A,B, lanes t1–t15 of Utp13). In all cases, cleavage of pre-rRNAs by MNase correlated with the appearance of smaller RNA fragments of similar size than the 5*′*ETS (Figure 2A,B, compare control lanes t0 and C15 with the other ones).

Cleavage by Utp10-MNase eliminated 35S, 23S, and 22.5S species (Figure 2A,B). We arbitrarily classified the lower part of northern blots in three regions (#1 to #3) to better describe the obtained RNA fragments. Since the band at #1c is present in all samples analyzed and recognized by probe bA0 but not A0A1 (Figure 2A,B), we assumed that it corresponds to the 5*′*ETS region. We detected a fragment slightly bigger than the 5*′*ETS region with probes bA0 and A0A1 in region #1 (Figure 2A,B, fragment #1b). Interestingly, the intensity of the band at #1b decreased during the time course, indicating further processing of this RNA fragment by Utp10-MNase. In agreement, smaller fragments were detected after 5 min of MNase activation in regions #2 and #3 (fragments #2a–c, and #3a). These RNAs might correspond to 5*′*ETS fragments containing the base-pairing site for probes bA0 and A0A1 (#2c) or only bA0 (#2a, #2b, and #3a), indicating multiple sensitive sites within the 5*′*ETS for Utp10-MNase. When RNA fragments were resolved in acrylamide gels, we detected a prominent band with a size similar to the U3 snoRNA (300 nucleotides) that increased in intensity during the time course (Figure 2C). Since this band was only detected with the bA0 probe (Figure 2C,D), it might correspond to the smallest fragment #3a observed in the northern blot from agarose gel (Figure 2A). The other RNA fragments detected in the agarose blot were not resolved in acrylamide, and they might overlap within the upper bands. It is striking that the signal observed in the RNA fragments appearing (at t1, t5, and t15) was stronger than expected from the initial signal detected in the pre-rRNAs (input, lanes I; and affinity-purified samples t0 and C15). Interestingly, we observed a large decrease in the background signal present in the lanes during the time course of the Utp10-MNase assay. As expected from the co-transcriptional association of Utp5 and Pwp2, the bait proteins should capture the nascent transcripts during the affinity purification. These RNAs were not resolved as single bands but smeared in the northern blots due to their different 3*′* ends. We interpret that MNase-induced cleavage produced defined 3*′* ends of the nascent transcripts, reducing the smear signal and resolving RNA fragments of similar size (see Figure 2A,B, compare t0 and C15 with the other lanes).

Structural probing with Nan1-MNase showed a slower kinetic when compared with Utp10-MNase. We observed several specific fragments produced by cleavage at 23S, 22.5S, and putative nascent pre-rRNAs. Since fragment #1a was detected with probes bA0 and A0A1, it should contain the 5*′*ETS region, including the A1 processing site. In addition, the fragment #1a is larger than #1b and #1c, which also contain the 5*′*ETS region. Thus, the fragment #1a might include the 5*′* region of 18S at the 3*′* end (Figure 2A,B). Furthermore, lower bands were similar to fragments obtained in the Utp10-MNase assay (Figure 2A,B, #1b and #2c), indicating the presence of hypersensitive sites for MNase common to Utp10 and Nan1 fusion proteins.

Structural probing with Utp12- and Utp13-MNase also showed cleavage of the 35S transcripts (Figure 2A,B). In contrast, the putative transcripts smearing in the lane and the 23S vanished preferentially in the samples containing Utp12-MNase compared with Utp13-MNase, indicating their specific cleavage by Utp12-MNase. Interestingly, we only observed a mild cleavage in the 35S pre-rRNA by Utp13-MNase, without any significant effect in the smearing transcripts or the 23S and 22.5S pre-rRNAs. Detailed analysis of fragments obtained by cleavage with Utp12-MNase showed the accumulation of rRNA fragments in region #1 recognized by probes bA0 and A0A1, suggesting a cleavage site in the 5*′* region of the 18S rRNA (fragment #1a). Moreover, we observed fragments in region #2 only recognized by probe bA0 (#2a). Strikingly, we observed a Utp12-MNase-specific fragment above the 18S region recognized by the A0A1 probe but not the bA0 one. Since this fragment does not include most of the 5*′*ETS, the result indicated that Utp12-MNase cleaved within the ITS1 or the 3*′*region of the 18S rRNA. As expected, we could not resolve any prominent band in the acrylamide blots due to the large size of the observed fragments.

**Figure 3:**
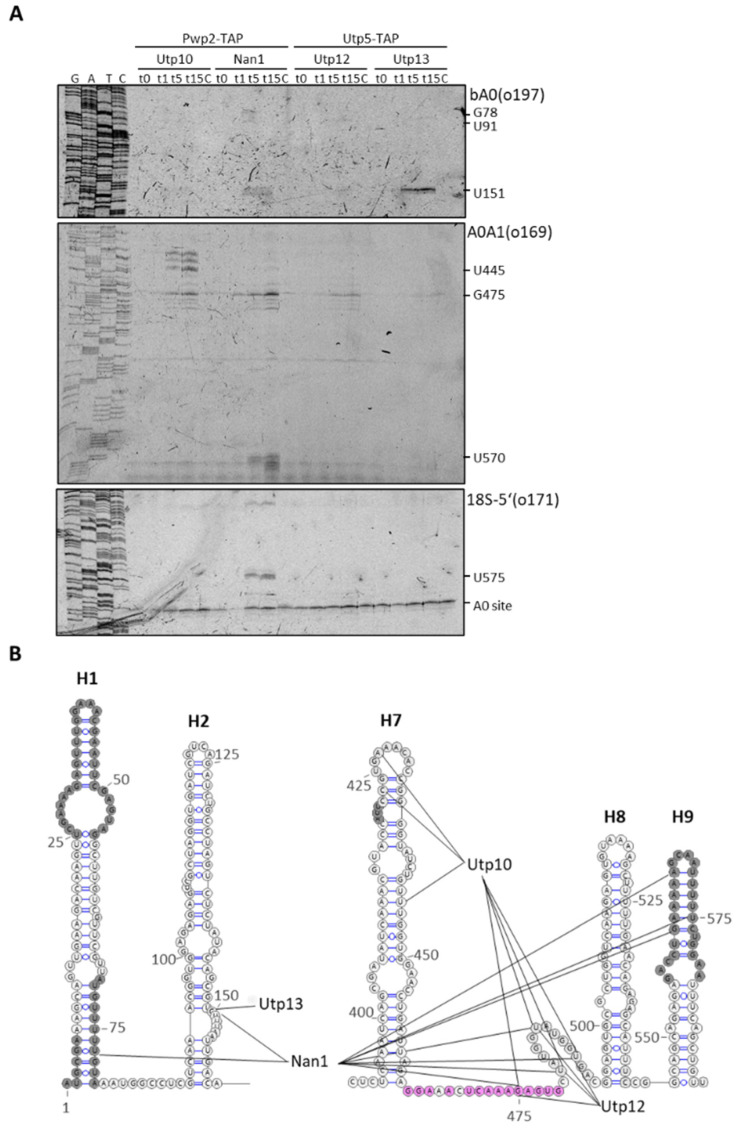
Characterization of RNA fragments obtained in the 5′ETS region after MNase treatment. Purified particles were incubated in the presence of CaCl_2_ for 10 min, and RNA fragments were obtained by hot phenol extraction and used for primer extension analysis with fluorescent oligonucleotides. (**A**) The 5′ETS region was explored using oligonucleotide base pairing in the 5′ETS (upper and middle panels) or the 5′ end of 18S rRNA (lower panel). (**B**) Stops in the reverse transcription observed in A and B were plotted in the schematic representation of the 5′ETS region (according to [22]). Components of the UTP complexes fused to TAP and MNase are indicated on top, and probes used for primer extension are indicated at the top right of the panels. Some nucleotides at prominent stops are indicated on the right side. Shaded nucleotides correspond to unresolved regions in published structures. Pink nucleotides correspond to the base-pairing site between helix 1b of the U3 snoRNA and the 5′ETS.

To determine the exact location of the hypersensitive sites detected by northern blotting within the 5*′*ETS, we performed primer extension analyses to map the 5*′*ends of obtained fragments. Using the bA0 probe, we could detect 5*′*ends for the fragments produced by Nan1-MNase (around G78) and Nan1-MNase and Utp13-MNase (around U151) (Figure 3A, upper panel). Using the A0A1 probe (Figure 3A, middle panel), we detected specific cleavage sites for Utp10 and Nan1 around U445 and U570, respectively. In addition, we observed common cleavage sites for Utp10, Nan1, Utp12, and Utp13 around G475, although they were more prominent for Utp10 and Nan1. RNA fragments ending around G475 lack the base-pairing site for the probe bA0. Since we did not observe these fragments in our northern blots (Figure 2A–C), they might correspond to 5*′*ends produced by the MNase on nascent transcripts not resolved because of their different 3*′*ends. Accordingly, the increase in the signal intensity in the primer extension reflects on the decreased intensity of the smear signal in the northern blots using probes bA0 and A0A1. Finally, using an oligo base-pairing the 5*′*end of 18S to analyze the last part of the 5*′*ETS (Figure 3A, lower panel), we reproduced the cuts observed around U570 and G475, although the last one with less sensitivity.

Altogether, the observed cuts for Utp10-MNase cluster within 75nts in the 5′ETS containing H7 and the base-pairing site for the snoRNA U3. Cleavage sites observed for Nan1-MNase spread through the 5′end. Finally, Utp12 and Utp13-MNase cleaved at more precise locations according to the weak cuts observed in the northern blot analyses.

### 2.2. Structural Probing of the ITS1 Region

**Figure 4:**
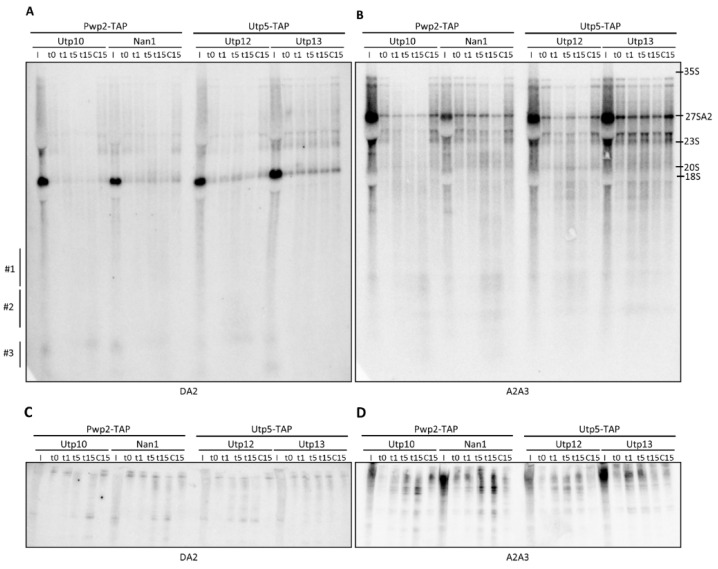
Structural probing of rRNA with tethered MNase analyzed by northern blot at the ITS1 region. Pwp2-TAP (for Utp10-MNase and Nan1-MNase) or Utp5-TAP (for Utp12-MNase and Utp13-MNase) containing pre-ribosomal particles were bound to IgG-Sepharose beads. After purification, MNase was activated by the addition of CaCl_2_, and samples were incubated at 16 °C during a time course. At indicated time points, the reaction was stopped. RNAs were obtained by hot-phenol extraction, resolved in either formaldehyde-agarose gels (**A**,**B**) or acrylamide gels (**C**,**D**), and transferred to membrane for northern blot analysis. Components of the UTP complexes fused to TAP or MNase are indicated on top, and probes used for the detection of RNAs in the northern blot are indicated below the panels. The localization of some prominent rRNAs is indicated on the right side. The three different regions (#1 to #3) to describe the observed RNA fragments in Figure 2 are also depicted for better comparison of the results.

To analyze the structural probing of the ITS1 region, we used probes DA2 and A2A3, which associate with different regions within the ITS1 (Figure 1A). DA2 probe allowed for the identification of 20S pre-rRNA in the input fractions but not in the affinity-purified fractions as expected (Figure 4A) [8]. An analysis of the cleavage patterns with the DA2 probe did not provide clear results in the agarose gel, which might be due to the high intensity of the 20S band. However, the weak signals obtained suggested cleavage of the 35S and the appearance of small RNAs smearing in region #3, when MNase was fused to Utp10, Nan1, or Utp12 (Figure 4A). An analysis of the ITS1 region with the A2A3 probe allowed us to identify the 35S, 27SA2, and 23S pre-rRNAs in the input and purified fractions (Figure 4B). Due to the high content of 27SA2 in cell extracts (compared signals of 27SA2 and 35S in input fractions), the 27SA2 used to be considered a contaminant when purified with assembly factors of the small subunit [31]. An analysis of RNAs with the A2A3 probe after cleavage induction showed a strong degradation of 35S by MNase fused to Utp10, Nan1, or Utp12 as previously observed with bA0 and A0A1. In addition, we also observed smears centered in spots localized in regions #1 and #2.

Consistently, the small RNA fragments were also detected in the blots obtained from acrylamide gels. The size of these fragments was similar to the 5S and 5.8S (Figure 4C,D) or to the U3 snoRNA (Figure 4D). As the fragments contained the base-pairing site for the indicated probe, we propose the localization of the cleavage sites within the ITS1 and/or the 3*′* end of the 18S rRNA (Figure 4C,D).

Despite the different kinetics, the size of fragments did not change substantially between the different MNase fusion proteins. However, 33S and 32S pre-rRNAs were less sensitive than 35S for MNase cleavage when fused to Nan1 and Utp12. These results are consistent with the preferential cleavage in the 3*′* end of the 5*′*ETS as previously outlined (Figure 2B), causing the accumulation of 33S/32S-like fragments.

Interestingly, the smear RNA signal fades in the upper part of the northern blot analysis of samples treated with MNase fused to Utp10, Nan1, and Utp12 but not in the lower parts. As mentioned before, this result might indicate the shortening of nascent transcripts with different 3*′* ends. According to our previous interpretation, this result suggests that MNase cleaves nascent transcripts within the region containing the 5*′*ETS and 18S when fused to the indicated factors.

**Figure 5:**
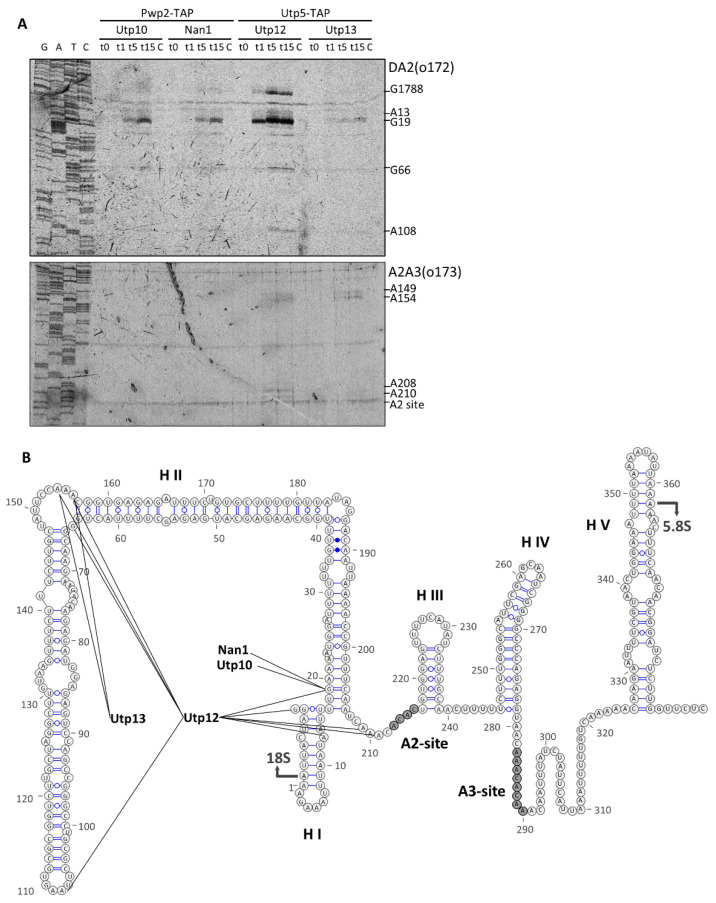
Characterization in the ITS1 region of RNA fragments obtained after MNase treatment. Purified particles were incubated in the presence of CaCl_2_ for 10 min, and RNA fragments were obtained by hot phenol extraction and used for primer extension analysis with fluorescent oligonucleotides. (**A**) The ITS1 region was explored using oligonucleotides that base pair in the ITS1 either between the cleavage sites D-A2 (upper panel) or between A2 and A3 (lower panel). (**B**) Stops in the reverse transcription observed in A and B were plotted in the schematic representation of the ITS1 region (according to [13]). Components of the UTP complexes fused to either TAP or MNase are indicated on top, and probes used for primer extension are indicated at the top right of the panels. Some nucleotides at prominent stops are indicated on the right side.

To identify the position of the MNase cleavage sites, we performed primer extension analysis of the obtained RNA fragments (Figure 5A). Using the probe DA2 base-pairing around the nucleotide 190 in the ITS1 (Figure 5B), we detected a common stop to all MNase-fusion constructs at the base of helix I (G19). We identified additional stops when MNase was fused to Utp12 with some of them concentrated around helix I, and others localizing in helix II. In contrast, by using probe A2A3 (base-pairing around the nucleotide 270), we could only detect stops when MNase was fused to either Utp12 or Utp13. Utp12-MNase produced cleavages at the base of helix I (A208 and A210) and in helix II (A149, A154), which were consistent with the previously defined cleavages. In the case of Utp13-MNase, the absence of cleavage around A208 was consistent with the weaker cleavage detected at G19, but we could attribute it to also cleavages at helix II (A149 and A154).

Altogether, our data indicate the proximity of the C-terminal domains (CTDs) of Utp10, Nan1, and Utp12 to the base of the helix I, which localizes near the A2 processing site. Moreover, the CTDs of Utp12 and Utp13 might localize in the proximity of helixes I and II, although the accessibility of Utp13-MNase to helix I seems to be somehow restricted.

### 2.3. Structural Probing of the U3 snoRNA

**Figure 6:**
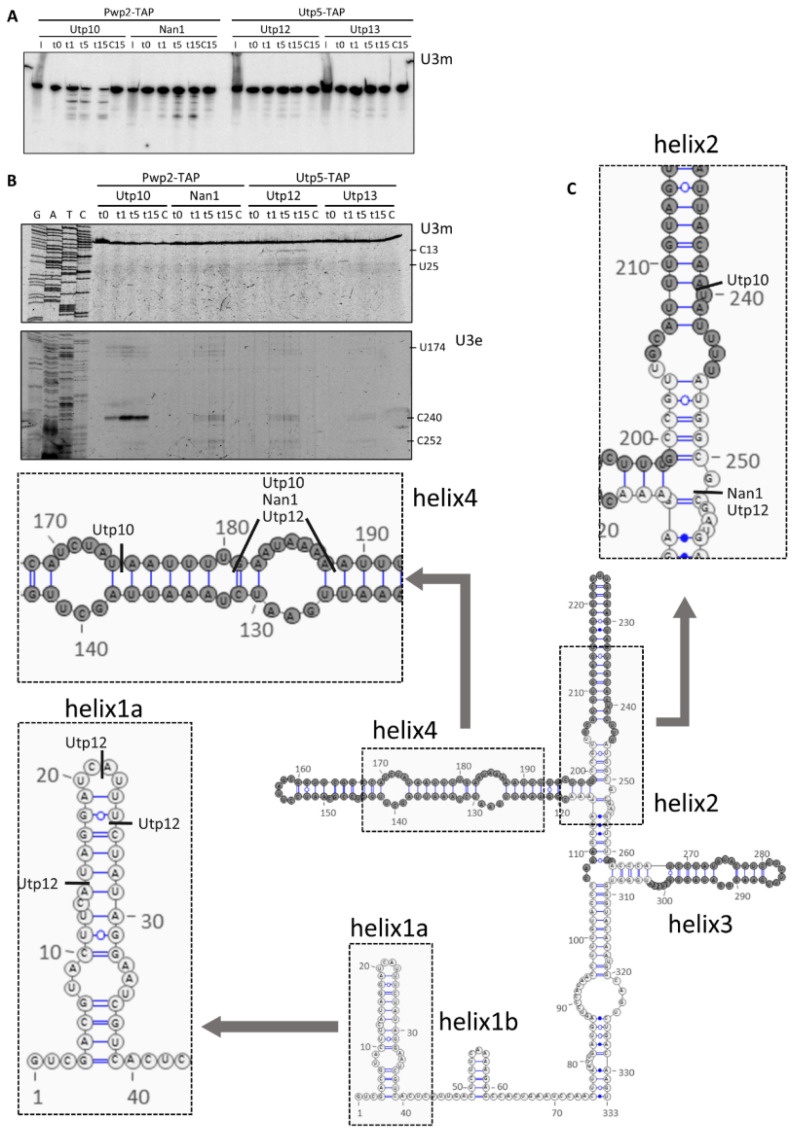
Structural probing of the U3 snoRNA with tethered MNase. Pwp2-TAP (for Utp10-MNase and Nan1-MNase) and Utp5-TAP (for Utp12-MNase and Utp13-MNase) containing pre-ribosomal particles were bound to IgG-Sepharose beads. After purification, MNase was activated by addition of CaCl_2_, and samples were incubated at 16 °C during a time course. At indicated time points, the reaction was stopped, and RNAs were obtained by hot-phenol extraction, resolved in acrylamide gels, and transferred to membrane for northern blot analysis (**A**) or explored by primer extension using fluorescent oligonucleotides (**B**). (**C**) Stops in the reverse transcription observed in B were plotted in the schematic representation of the U3 snoRNA (according to [14,15]). Components of the UTP complexes fused to either TAP or MNase are indicated on top, probes used for detection of RNAs in the northern blot are indicated below the panels, and probes used for primer extension are indicated at the left of the panels. Some nucleotides at prominent stops are indicated on the right side.

To characterize the proximity between the CTD of the Utps and the U3 snoRNA, we hybridized the acrylamide northern blot with a probe specific for the U3 snoRNA (Figure 6A). MNase tethered to the CTD of Utp10 produced RNA fragments between 250 and 150 nucleotides containing the region 139–153 of the U3 snoRNA (base-pairing of the oligonucleotide probe in the northern blot analysis). In contrast, MNase tethered to Nan1 produced a different pattern of U3 fragments but with similar lengths. These fragments are dependent on the MNase activity since they were not detected in the control lanes. Moreover, Nan1 and Utp10 tethered MNase produced fragments of different sizes due to the different positions of the MNase. Interestingly, only low intense bands were detected when either Utp12 or Utp13 were tethered with MNase, indicating a mild cleavage of the U3 snoRNA. These results indicate higher proximity and accessibility of U3 domains to the CTDs of Nan1 and Utp10 compared with the CTDs of Utp12 and Utp13.

To characterize the cleavage of MNase in the U3 snoRNA in more detail, we performed a primer extension analysis (Figure 6B) with oligonucleotides base-pairing at either the middle region (upper panel) or the 3*′*end of the U3 snoRNA (lower panel). The identified stops in the reverse transcription indicated cleavage in the helices 2 and 4 of the U3 snoRNA by Utp10 tethered MNase (Figure 6B,C). Interestingly, these regions are not visualized in the cryo-EM models for the small ribosomal subunit processome [18,19,20,21,22,32]. For Nan1 tethered MNase, we could also identify stops sites within helices 2 and 4 (Figure 6B,C). However, the weaker intensity and the additional cleavage at C252 suggest a slightly different orientation of the CTD of Nan1. As for Nan1, only mild signals were obtained for MNase fused to Utp12 and Utp13 in helices 2 and 4 (Figure 6B lower panel and Figure 6C). However, Utp12-MNase showed additional stops within helix 1a (Figure 6B upper panel and Figure 6C).

Altogether, our results suggest a large exposed surface of U3, which results in hypersensitive sites located in the proximity of the CTDs of Utp10. We speculate that the weaker signals detected in the cases of Nan1, Utp12, and Utp13 might reflect either a higher distance or a lower population of pre-ribosomal particles with the U3 snoRNA exposed to the attached MNase.

## 3. Discussion

In contrast with the CRAC or CLIP methods [33,34], our approach does not aim to resolve contacts between protein and RNA but to provide three-dimensional information about RNA folding around tethered MNase [23]. We identified several hypersensitive sites for MNase within the 5′ETS. These cleavage sites are specific for the assembly factor fused to the MNase and consistent with the expected position of their C-terminal domains (CTDs). Our dataset complements high-resolution structural data since we detected several cleavage sites in unresolved regions of the 5′ETS, ITS1, and U3 snoRNA [18,19,20,21,22,32]. In agreement with our interpretation, some of these regions should be solvent-exposed sequences [18,19,22]. Discrepancies between our dataset and published CRAC data are explained because MNase fusion proteins probe for RNA environment [23,35]. Moreover, our data suggest the position of the CTD of Utp10 complementing the absence of more than 1300 amino acids (75% of the protein) in the available models [18,19,22]. Nevertheless, we cannot disregard that the observed MNase-cleavages might result from different folding states of pre-ribosomal precursors or may reflect the flexibility of small ribosomal subunit (SSU) processome components during the assembly of ribosomes, as recently proposed [20,21]. Structural probing analyses of pre-ribosomal particles arrested in their maturation by depleting specific assembly factors (AFs) might help to address these questions.

**Figure 7:**
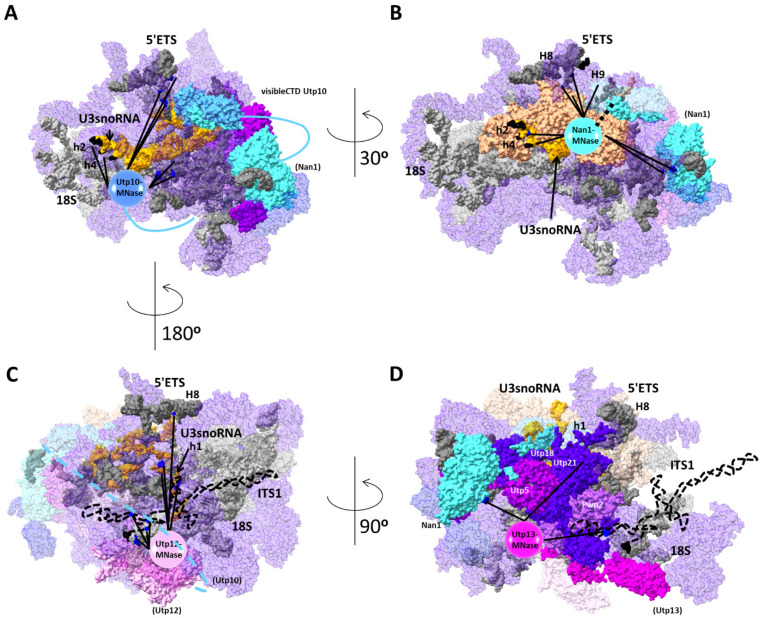
Prediction for localization of 5′ETS, ITS1, h4, and h5 of the U3snoRNA, and the CTD of proteins Nan1, Utp10, Utp12, and Utp13. Cleavages observed by tethered MNases to Utp10 (**A**), Nan1 (**B**), Utp12 (**C**), and Utp13 (**D**) are depicted. A circular form representing the MNase is placed closed to the cleavage sites observed for Utp10 or near the CTDs of Nan1, Utp12, and Utp13. Cleavage sites are indicated by lines and blue dots. The 5′ and 3′ end of RNA regions not visualized in cryo-EM structures are depicted in black, and not visualized regions are represented by dashed lines. Relevant elements are colorized as follows: (**A**) 5′ETS (light gray), 18S (dark grey), U3 snoRNA (yellow), Nan1 (cyan), Utp10 (light blue), Utp5 (violet), Pwp2 (orchid), and SSU-processome components (violet with transparency); (**B**) same as (**A**) and U3 snoRNP (wheat); (**C**) same as (**A**) and Utp12 (pale pink); and (**D**) same as (**A**) and Utp13 (fuchsia), Utp21 and Utp18 (purple). The name of relevant RNA helixes is indicated in uppercase for pre-rRNA and lowercase for the U3 snoRNA. Rotation of particles is indicated. Other proteins relevant for the interpretation are also depicted. Figures modified from pdb:5WLC, [18].

The 5′ETS region near the A0 cleavage site, and the tips of H4 and H7 were localized close to each other within the SSU-processome [18,19] (Figure 7). Our structural probing with the Utp10 tethered MNase indicates the position of the CTD of Utp10 near these sites (Figure 7A). On the other hand, the structural probing with Nan1 tethered MNase is also consistent with structure data for the localization of H9 [20,21]. The CTDs of both proteins might localize nearby, as they share several cleavage sites at 5′ETS and U3 snoRNA. In agreement with our results, Nan1 and Utp10 contact the 3′ end of H1 and the 5′ side of H2, respectively, indicating their physical proximity [35]. However, both CTDs would only be close to each other if the repetitive helical domain of Utp10 enclosed the SSU-processome as it was observed in low-resolution structures (Figure 7A,B) [32]. Interestingly, several contact sites between Utp10 with the 5′ETS and the U3 snoRNA cannot be explained from the published structures [35]. We think our model integrates these contacts of Utp10 with its extreme flexibility, providing further information for the chaperoning role of t-UTP complex on the SSU-processome [18,35,36].

Altogether, our results would indicate that Utp10 clamps the t-Utp complex within the SSU-processome with its CTD reaching the other side of the particle near the U3 snoRNA. We propose that the folding of the Utp10 improves the stability of the SSU-processome during assembly. In addition, the proximity of the CTDs of Nan1 and Utp10 might fix the position of the 5′ETS, localizing the A0 and A1 processing sites on the other side of the SSU-processome near Utp12 and Utp13. In agreement, MNase fused to the CTD of Utp12 and Utp13 cleaved at the 5′ region of the 18S.

Regarding the ITS1, it is preferably located in the proximity of the CTDs of Utp12 and Utp13 (Figure 7C,D). This interpretation is consistent with the physical proximity of their CTDs [18,19,20,21,22,32]. Interestingly, the strain expressing Utp12 fused to MNase was the only one showing a mild growth defect. Since the CTD of Utp12 is buried within the SSU-processome, the MNase might create a steric hindrance interfering with the folding of the pre-rRNA or the association of AFs and ribosomal proteins. Although the ITS1 localizes far away from the CTDs of Nan1 and Utp10, the specific cleavages indicate their proximity in some stage of pre-ribosomal maturation. On one side, recent data shows a structural rearrangement occurring after cleavage at the A1-site in the 5′ETS that may explain the transient proximity between ITS1 and the CTDs of Nan1 and Utp10 [37]. As an alternative, the proximity between the ITS1 and the CTDs of Utp10 and Nan1 might occur during SSU-processome folding to clamp the ITS1 between the t-UTP and UTP-B complexes (Figure 7C,D). We suggest that positioning the ITS1 within the complex t-UTP and UTP-B fixates the position of the A2 cleavage site and creates the binding platform for the endonuclease. This interpretation integrates the high conservation of the cleaved sequence with the sequence-independent cleavage occurring at the A2 site during the maturation of pre-ribosomal particles [38,39].

Altogether, our results localized A0, A1, and A2 cleavage sites close to each other. Although the endonuclease of the A0 site remains elusive, Utp24 might be the endonuclease of at least A1 and A2 sites [40,41]. By bringing together all these sites, our model would support the stochastic cleavage at A0, A1, and A2 sites and the short life suggested for 22.5, 22S, and 21.5S rRNA precursors [8].

Interestingly cleavage sites induced by Nan1-MNase at G475, U481, U486, and U491 of 5′ETS clustered close to the cleavage sites at the U3 snoRNA. However, the protein components of the U3 snoRNP should protect these sites (Figure 7A,B). Therefore, either the CTD of Nan1 is trapped inside the SSU-processome or the cleavages took place before the complete assembly of the SSU-processome. However, other cleavage sites localized in exposed residues of U3, indicating the external localization of the CTD of Nan1. Moreover, CRAC data also localize Utp10 and Nan1 in the proximity of helices 2 and 4 of the U3 snoRNA [35]. Since these contacts cannot be explained from the published structures, we think they are consistent with our proposed location for the CTD of Utp10.

According to the expected position for the CTD of Utp12, we observed cleavages at the helix 1a of the U3snoRNA within the unpaired nucleotides. Since this region is occluded by Mpp10, these cleavages may occur before its association. Due to the growth defect of the strain harboring the Utp12-MNase fusion, we cannot exclude that the addition of MNase delays the association of Mpp10. Nevertheless, structural probing analysis of pre-ribosomal particles accumulated in the absence of SSU-processome components with tethered MNase would provide mechanistic details for the assembly of the SSU-processome.

The most striking result concerns the bleaching of the lanes in the northern blot after activation of the MNase. As indicated, we think that the background signal detected in the northern blot analysis corresponds to the nascent pre-rRNA transcripts previously described [1,2]. Nascent transcripts processed post-transcriptionally account at least for 30% to 50% of the total transcripts, and the absence of a common 3′ end does not allow for their separation as single bands. Structural probing with the MNase provides a common 3′ end to non-terminated transcripts that share a similar folding state, and it allows for resolving a significant population of pre-rRNAs that has never been considered before. Therefore, new technical strategies should be developed for the characterization of nascent pre-ribosomal particles. Structural probing analysis of pre-ribosomal particles with tethered MNase would be a feasible alternative to investigate the folding and flexibility of the nascent pre-rRNAs.

## 4. Materials and Methods

Yeast strains and microbiological procedures: the oligonucleotides, plasmids, yeast strains, and antibodies used in this work are listed in Tables S1–S4. Modified yeast strains (Table S3) were obtained by homolog recombination using PCR-amplified cassettes from plasmids pYM-TAP::URA3 or K2132 using the standard S2 and S3 sequences [34]. Plasmid pYM-TAP::URA3 was created by cloning the fragment containing the coding sequences for TAP and URA3 amplified with oligonucleotides #3883 and #3884 from pBS1539. Plasmid K2132 was created by cloning the SalI fragment (300 bp aprox) obtained from plasmid K2116 in the plasmid pKM9 [42]. This strategy eliminates the frameshift existing in pKM9 to use oligonucleotides designed for amplification of other gene cassettes [28]. Yeast cells were cultured in YPD (1% yeast extract, 2% bactopeptone, and 2% glucose).

Cell lysis for affinity purification. The cell pellets corresponding to 500 mL of yeast culture at the exponential growth phase were washed in 10 mL Buffer P1G (150 mM KAc, 20 mM Tris, pH 8.0, 5 mM MgCl2, 1 mM DTT, 0.2% (*w*/*v*) Triton, and 5 mM EGTA) supplemented with Protease Inhibitors (PIs, 1mM PMSF, and 2mM Benzamidine). Cell pellets were mixed with 1.5 volumes (g/mL) of Buffer P1G containing PIs and RNasin (Promega), and cell suspensions were mixed with 1.4 g glass beads (diameter: 0.75–1.0 mm). Cell disruption was performed at 4 °C for 6 × 30 s at 6000 rpm with 5× 30 s pausing in Precellys Evolution coupled to Cryolys (Bertin Instruments). Cell lysates were clarified by centrifugation at 18,000× *g* for 15 min at 4 °C.

Affinity purification using IgG-coupled Sepharose beads. Affinity purification was performed as described in [8] using Buffer P1G. Washed beads were finally resuspended in 6 mL Buffer P1G, and six aliquots containing equal amounts of beads were prepared. The protein content of one aliquot of beads was eluted by incubation at 95 °C for 5 min with 1x SDS Gel loading buffer [43] and analyzed by SDS-PAGE and western blotting as described below. The remaining five aliquots were used for the MNase assay as described below.

MNase assay on purified fractions: five aliquots of beads containing affinity purified complexes were resuspended in 200 µL Buffer P1G. An MNase assay was initiated by the addition of CaCl_2_ to obtain a final concentration of 10 mM CaCl_2_, and samples were incubated at 16 °C. The reaction was stopped at different times for each aliquot, either 1, 5, 10, or 15 min (t1, t5, t10, and t15, respectively) by adding 500 µL cold AE+ Buffer (50 mM NaAc, pH 5.3, 10 mM EDTA, pH 8.0, 10 mM EGTA, pH 8.0). Control samples t0 and c15 were supplemented with Buffer P1G instead of CaCl_2_ and mixed with 500 µL cold AE+ Buffer either immediately (t0; 0 min) or after 15 min (C15) incubation at 16 °C. Downstream RNA analysis was performed as described below.

SDS-PAGE and western blotting. Samples for SDS-PAGE analysis were mixed with 1x SDS Gel loading buffer and processed [43]. About 0.2% of whole-cell lysates (W) and flow-through (F), 2% of wash (w), and 10% of eluates (Ip) were resolved in 8% SDS-PAGE. Proteins were transferred to PVDF membranes, and detection of proteins was performed with antibodies summarized in Table S4. Protein signals were visualized using the Chemiluminescence western blotting reagent (Roche) in a LAS-3000 device (Fujiflm).

RNA extraction and northern blotting: RNAs were extracted from whole-cell lysates and bead samples after MNase assay using the hot acidic phenol/chloroform method [44]. Approximately 0.2% of whole-cell lysates (I) and 20% of each MNase sample were analyzed by northern blotting. Northern blotting analysis after RNA separation on denaturing urea polyacrylamide gels or formaldehyde/MOPS agarose gels was carried out as described [43]. The detection of membranes with 32P-labeled probes (listed in Table S1) was performed as described previously [5].

Primer extension of purified RNAs after MNase treatment using fluorescent oligonucleotides; 500 ng of purified RNAs from the MNase control sample (t0) and from samples treated for 10 min (t10) with MNase were subjected to primer extension analysis as previously described [45]; and 1 pmol of the fluorescent labeled primers listed in Table S1 were used to synthesize cDNA. Instead of EtOH precipitation of hydrolyzed cDNA, pH of samples was adjusted by adding HCl to a final concentration of 80 mM. Samples were mixed with an equal volume of stop buffer (95% deionized formamide, 15 mM EDTA, pH 8.0, and bromine phenol blue). Sequencing reactions using 2 pmol of the same fluorescent labeled primers were performed with the Thermo Sequenase cycle Sequencing Kit (Thermo Fisher Scientific) using the plasmid K375 (Table S2) encoding the rDNA locus of *S. cerevisiae* as a template; 16% of the cDNA samples and 1 µL of each sequencing reaction were mixed with 3 µL of the stop solution and resolved in the 14% polyacrylamide gel containing 6 M urea in 1x TBE [46]. Electrophoresis was performed for 3.5 h at 6 W and constant temperature between 50 and 55 °C. Wet gels were detected using LI-COR Odyssey IR Imaging System.

## Data Availability

Not applicable.

## Supplementary Materials

The following are available online at https://www.mdpi.com/article/10.3390/ncrna8010001/s1, Figure S1: Tethering of MNase to RBFs for structural probing. Table S1: Oligonucleotides used in the study, Table S2: Plasmids used in the study, Table S3: Yeast strains used in the study, Table S4: Antibodies used in the study.
