# Supplementary material for: Structural Probing with MNase Tethered to Ribosome Assembly Factors Resolves Flexible RNA Regions within the Nascent Pre-Ribosomal RNA
Source: Noncoding RNA. 2022 Jan 9;8(1):1. doi: 10.3390/ncrna8010001 (PMC8788456; doi:10.3390/ncrna8010001)
Supplement: Supplementary file 1 [file ncrna-08-00001-s001.zip › Supplementary Figure 1.pdf]

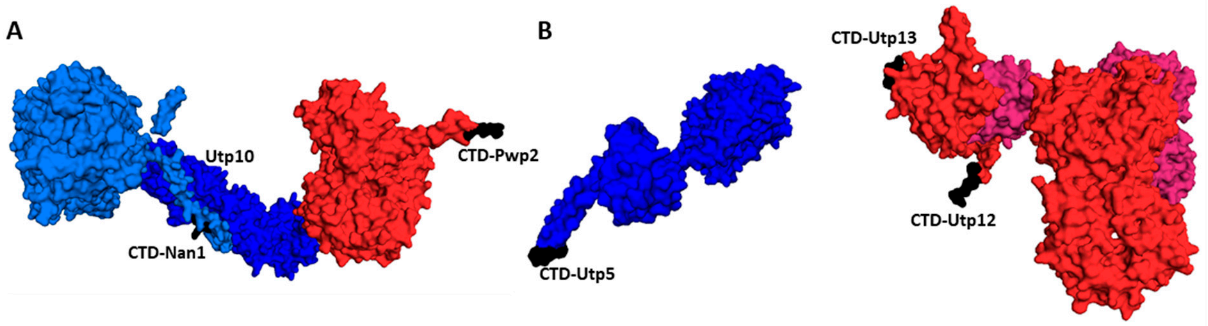

Supplementary Figure S1. Tethering of Mnase to RBFs for structure probing. (**A**) Detail for the localization of the CTD (black densities) of Nan1 (light blue) and Pwp2 (red); note that only the first 400 aa of Utp10 were modelled (pdb:5WLC, Barandun et al., 2017). (**B**). Detail for the localization of the CTD (black densities) of Utp5 (blue), Utp12 (red), and Utp13 (pdb:5WLC, Barandun et al., 2017).
