# Supplementary material for: Structural Probing with MNase Tethered to Ribosome Assembly Factors Resolves Flexible RNA Regions within the Nascent Pre-Ribosomal RNA
Source: Noncoding RNA. 2022 Jan 9;8(1):1. doi: 10.3390/ncrna8010001 (PMC8788456; doi:10.3390/ncrna8010001)
Supplement: Supplementary file 1 [file ncrna-08-00001-s001.zip › Supplementary Tables.pdf]

Suplementary Tables

Table S1: Oligonucleotides used in this study. Oligonucleotides used for different purposes are described. Database numbers, sequence and usage are indicated. Oligonucleotides marked with "#" belong to the Department of Biochemistry III at the University of Regensburg. Oligonucleotides marked with "o" belong to the collection of the UTP-Lab.

Col. N.	Name	5′-3′-sequence	Purpose
**o54**	Pwp2_S3	GATTCCAGTGATGAAGAAGA AAATGAGAAAGAGCTTCCTC GTACGCTGCAGGTCGAC	Amplification of *HA-MNase::KANMX* cassette (o54-#3941)
**o55**	Utp12_S3	GAGTAATAGTGCAAGGAAGA GAGTTTTCGGGACCGTTATAC GTACGCTGCAGGTCGAC	Amplification of *HA-MNase::KANMX* cassette (o55-o56)
**o56**	Utp12_S2	GGGCAGAGTAATGCCGGTATT ATGTAGGATAATAGTTTTAAT CGATGAATTCGAGCTCG	Amplification of *HA-MNase::KANMX* cassette (o55-o56)
**o57**	Utp13_S3	CATATTAGACTACGCGCTAGT GGAAATGGATAAGCTATTCCG TACGCTGCAGGTCGAC	Amplification of *HA-MNase::KANMX* cassette (o57-o58)
**o58**	Utp13_S2	GCCTTGCTTTTAAAGGCTCAA TATCTTGGACTTACTGCATCG ATGAATTCGAGCTCG	Amplification of *HA-MNase::KANMX* cassette (o57-o58)
**o59**	Utp18_S3	CGAACAAGGTAAAGTGAGGC TCTGGAAATTAAACCACTACC GTACGCTGCAGGTCGAC	Amplification of *HA-MNase::KANMX* cassette (o59-o60)
**o60**	Utp18_S2	GTACATTGCTTTTAATATCTA AGTTTGTCTATAAATGGAAAT ATCGATGAATTCGAGCTCG	Amplification of *HA-MNase::KANMX* cassette (o59-o60)
**o61**	Utp6_S3	CAGAAGATGATCCTAGGTAT AAAATTCTAGATTTAATTATC AGCAAACTTCGTACGCTGCAG GTCGAC	Amplification of *HA-MNase::KANMX* cassette (o61-o62)
**o62**	Utp6_S2	CTGATATGTAATTGCATTTAT AAAATGTACAGTACCGCATAT CGATGAATTCGAGCTCG	Amplification of *HA-MNase::KANMX* cassette (o61-o62)
**o67**	bA0	CGCTGCTCACCAATGG	hybridizes in the rDNA, 5′ETS before A0 site
**o169**	2921-A0A1	(DY682)CCCACCTATTCCCTCT TGCTAG	Fluorescent oligonucleotide for primer extension. Hybridization site at 5′ETS between A0 and A1 sites
**o171**	205-A1-18S	(DY682)CATGGCTTAATCTTTG AGAC	Fluorescent oligonucleotide for primer extension. Hybridization site at 5′ of 18S sequence
**o172**	206-D/A2	(DY682)CGGTTTTAATTGTCCT A	Fluorescent oligonucleotide for primer extension. Hybridization site at ITS1 between D and A2 sites
**o173**	207-A2/A3	(DY682)TGTTACCTCTGGGCCC	Fluorescent oligonucleotide for primer extension. Hybridization

			site at ITS1 between A2 and A3 sites
**o177**	SnR17-Middle3468	(DY682)CCGCTAAGGATTGCGGACCAAGC	Fluorescent oligonucleotide for primer extension. Hybridization site at snR17A (U3 snoRNA)
**o197**	vA0(DY682)	[DY682]CGCTGCTCACCAATGG	Fluorescent oligonucleotide for primer extension. Hybridization site at 5′ETS before A0 site
**o198**	5'18S_rev(DY682)	[DY682]AACGATAACTGATTTAATGAGCC	Fluorescent oligonucleotide for primer extension. Hybridization site at 5′ of 18S sequence
**o205**	snr17a_END	(DY682)TCAGACTGCCATTTGTACCC	Fluorescent oligonucleotide for primer extension. Hybridization site at snR17A (U3 snoRNA) at the 3′end
**#206**	o3-D/A2	CGGTTTTAATTGTCCTA	hybridizes in the rDNA, ITS1 between D and A2 sites
**#207**	o4-A2/A3	TGTTACCTCTGGGCCC	hybridizes in the rDNA, ITS1 between A2 and A3 sites
**#2921**	A0A1-sonde	CCCACCTATTCCCTCTTGCTAG	hybridizes in the rDNA, 5′ETS between A0 and A1 sites
**#3468**	SnR17-Middle	CCGCTAAGGATTGCGGACCAAGC	hybridizes in the U3 snoRNA
**#3469**	SnR30-Middle	CAGAGGAGAAGTCAGGAGCTGC	hybridizes in the snR30 snoRNA
**#3692**	NAN1-S3-fw	GTGCAAATGGATACATTTTTGATCGTGTGATGAAAGTATTAACACGTACGCTGCAGGTCGAC	Amplification of *HA-MNase::KANMX* cassette (#3692-#3693)
**#3693**	NAN1-S2-rev	ATACCTTTATATCTATTTTGGATATCCAATGGAAGAACCCTAAAGATCGATGAATTCGAGCTCG	Amplification of *HA-MNase::KANMX* cassette (#3692-#3693)
**#3694**	UTP4-S3-fw	TTTCACTCCAAACAAAAGGCGTTTATTCAACCAAAGTTAGTGTTTCGTACGCTGCAGGTCGAC	Amplification of *HA-MNase::KANMX* cassette (#3694-#3695)
**#3695**	UTP4-S2-rev	TTTTAATAGCATCTCTCTATTCTTCGGTATGTTGACTTAAATTAAATCGATGAATTCGAGCTCG	Amplification of *HA-MNase::KANMX* cassette (#3694-#3695)
**#3696**	UTP5-S3-fw	AGCGACGGCGAGGAGGAAGCCGGATATAGTGACGTTGAGATGGAACGTACGCTGCAGGTCGAC	Amplification of *HA-MNase::KANMX* and TAP::URA3 cassettes (#3696-#3697)
**#3697**	UTP5-S2-rev	ATTTTTGTATTCTGATGCGTGAAAGCATTTTATGCATGATATCCTATCGATGAATTCGAGCTCG	Amplification of *HA-MNase::KANMX* and TAP::URA3 cassettes (#3696-#3697)
**#3698**	UTP8-S3-fw	CAGAAGCGAGCCTTACCCACTTACACCATGGAATACTTGGACATTCGTACGCTGCAGGTCGAC	Amplification of *HA-MNase::KANMX* cassette (#3698-#3699)

**#3699**	UTP8-S2-rev	TTTTCTATATAGGTATTATACAATACAATCAAATTCATTGCATACATCGATGAATTCGAGCTCG	Amplification of *HA-MNase::KANMX* cassette (#3698-#3699)
**#3700**	UTP9-S3-fw	AATGGAGAAAGTGACGAATTTGTCGACGCATCGGAATACAAAGATCGTACGCTGCAGGTCGAC	Amplification of *HA-MNase::KANMX* cassette (#3700-#3701)
**#3701**	UTP9-S2-rev	TGAAACTTTTCTTTGTATATACTTAAATATATTTAAAATGCTTGAATCGATGAATTCGAGCTCG	Amplification of *HA-MNase::KANMX* cassette (#3700-#3701)
**#3702**	UTP10-S3-fw	GTTGTTGAAAACGTTTTAGGGGAACCTTTTGATAGGTATTTAGATCGTACGCTGCAGGTCGAC	Amplification of *HA-MNase::KANMX* cassette (#3702-#3703)
**#3703**	UTP10-S2-rev	GTGTTTTACTTTACAAAAATTTACATAATACTTCACCTTTTTTTTATCGATGAATTCGAGCTCG	Amplification of *HA-MNase::KANMX* cassette (#3702-#3703)
**#3704**	UTP15-S3-fw	AAAGAAGCTCAAAGAATTGAAGGTATGCTTCAACTTTTAACGAGTCGTACGCTGCAGGTCGAC	Amplification of *HA-MNase::KANMX* cassette (#3704-#3705)
**#3705**	UTP15-S2-rev	ATCTATAAAATAGATACAAATTAAAATCTATATGCTAATATTTCCATCGATGAATTCGAGCTCG	Amplification of *HA-MNase::KANMX* cassette (#3704-#3705)
**#3883**	S3_TAP	CAGCTGAAGCTTCGTACGCTGCAGGTCGACATGGAAAAGAGAAGATGGAAAAAG	Amplification of *TAP::URA3* (#3883-#3884)
**#3884**	S2_pBS	GGATCTGATATCATCGATGAATTCGAGCTCGATACGACTCACTATAGGGCG	Amplification of *TAP::URA3* (#3883-#3884)
**#3941**	Pwp2_S2Ct	ATGTGAATGTGCATAAATAGAGGACAGTGAATATTTTTCAATCGATGAATTCGAGCTCG	Amplification of *HA-MNase::KANMX* cassette (o54-#3941)

Table S2: Plasmids used in this study. Database numbers, name of the plasmid, contained markers for selection and the cloning procedure are indicated. Plasmids marked with "#" belong to the Department of Biochemistry III at the University of Regensburg. Plasmids marked with "p" belong to the collection of the UTP-Lab.

#	**Name**	**Gene**	**Marker**	**Source**	**Cloning procedure**
**p96**	pYM-TAP::URA3	*TAP::URA3* cassette	Amp	UTP-Lab	URA3 from pBS1539 cloned in pYM15 (Janke et al., 2004)
**#97**	pBS1539	*TAP::URA3* cassette	Amp	BCIII	[25]
**#643**	pKM9	*MNase-3xHA::KANMX* cassette	Amp	BCIII	
**#2116**	K2116	*LongHelix-MNase*	Amp	BCIII	Gene synthesis by Entelechon
**#2132**	K2132	*MNase-3xHA::KANMX* cassette	Amp	BCIII	This work. See Methods

Table S3: Yeast strains used in this study. Database numbers, name of the strain, genotype, and source are indicated. Strains labeled with "#" belong to the Department of Biochemistry III at the University of Regensburg. Strains labeled with "Y" belong to the collection of the UTP-Lab.

#	**Name**	**Genotype**	**Source**
**#206**	BY4741	MAT a; *his3-1; leu2-0; met15-0; ura3-0*	Biochemie III
**#207**	BY4742	MAT a; *his3-1; leu2-0; ura3-0*; lys2-0	Biochemie III
**#2583**	Y2583	*MAT a; his3-1; leu2-0; met15-0; ura3-0; YML063w::KANMX4; YLR441C::HIS3; YDR324C::UTP4-TAP::klURA3; YEp-P_{RPS28}::2xHA-MNase-RPS1*	Biochemie III
**#2864**	Y2864	MAT α; *his3-1; leu2-0; lys2-0; ura3-0; YCR057C::PWP2-TAP::URA3; YDR324C::KANMX::MNase-3xHA-UTP4*	This study
**#2866**	Y2866	MAT α; *his3-1; leu2-0; lys2-0; ura3-0; YCR057C::PWP2-TAP::URA3; YDR398W::KANMX::MNase-3xHA-UTP5*	This study
**#2868**	Y2868	MAT α; *his3-1; leu2-0; lys2-0; ura3-0; YCR057C::PWP2-TAP::URA3; YGR128C::KANMX::MNase-3xHA-UTP8*	This study
**#2870**	Y2870	MAT α; *his3-1; leu2-0; lys2-0; ura3-0; YCR057C::PWP2-TAP::URA3; YHR196W::KANMX::MNase-3xHA-UTP9*	This study
**#2873**	Y2873	MAT α; *his3-1; leu2-0; lys2-0; ura3-0; PWP2-TAP::URA3; YJL109C::KANMX::MNase-3xHA-UTP10*	This study
**#2874**	Y2874	MAT α; *his3-1; leu2-0; lys2-0; ura3-0; YCR057C::PWP2-TAP::URA3; YMR093W::KANMX::MNase-3xHA-UTP15*	This study
**#2976**	Y2976	MAT α; *his3-1; leu2-0; lys2-0; ura3-0; YCR057C::PWP2-TAP-URA3; YPL126W::KANMX::MNase-3xHA-NAN1*	This study
**Y319**	YFAT1-1a	MATa; *his3-1; leu2-0; met15-0; ura3-0; YCR057C::KANMX::MNase-3xHA-PWP2*	This study
**Y321**	YFAT2-1a	MATa; *his3-1; leu2-0; met15-0; ura3-0; YLR409C::KANMX::MNase-3xHA-UTP21*	This study
**Y323**	YFAT3-1a	MATa; *his3-1; leu2-0; met15-0; ura3-0; YLR129W::KANMX::MNase-3xHA-UTP12*	This study
**Y325**	YFAT4-1a	MATa; *his3-1; leu2-0; met15-0; ura3-0; YLR222C::KANMX::MNase-3xHA-UTP13*	This study
**Y327**	YFAT5-1a	MATa; *his3-1; leu2-0; met15-0; ura3-0; YDR449C::KANMX::MNase-3xHA-UTP6*	This study
**Y329**	YFAT6-1a	MATa; *his3-1; leu2-0; met15-0; ura3-0; YJL069C::KANMX::MNase-3xHA-UTP18*	This study

**Y368**	YMCT2-1a	MATa; *his3-1*; *leu2-0*; *met15-0*; *ura3-0; YDR398W::UTP5-TAP::URA3; YLR129W::KANMX::MNase-3xHA-UTP12*	This study
**Y370**	YMCT3-1a	MATa; *his3-1*; *leu2-0*; *met15-0*; *ura3-0; YDR398W::UTP5-TAP::URA3; YLR222C::KANMX::MNase-3xHA-UTP13*	This study

Table S4: Antibodies used in this study.

Antibody	Origin	Dilution	Origin
**Anti-PAP (peroxidase conjugated)**	rabbit	1:5000	GenScript
**Anti-Utp18**	rabbit	1:1000	Agro-Bio
**Anti HA**	rat (3F10)	1:5000	Roche
**Anti-rat (peroxidase conjugated)**	goat	1:5000	Dianova
**Anti-rabbit (peroxidase conjugated)**	goat	1:5000	Dianova
